# Environmental Health Related Socio-Spatial Inequalities: Identifying “Hotspots” of Environmental Burdens and Social Vulnerability

**DOI:** 10.3390/ijerph13070691

**Published:** 2016-07-09

**Authors:** Rehana Shrestha, Johannes Flacke, Javier Martinez, Martin van Maarseveen

**Affiliations:** Faculty of Geo-information Science and Earth Observation (ITC), University of Twente, PO Box 217, 7500 AE Enschede, The Netherlands; j.flacke@utwente.nl (J.F.); j.martinez@utwente.nl (J.M.); m.f.a.m.vanmaarseveen@utwente.nl (M.M.)

**Keywords:** multiple burdens and benefits, spatial inequality, social vulnerability, “hotspots”, indicators, dasymetric method

## Abstract

Differential exposure to multiple environmental burdens and benefits and their distribution across a population with varying vulnerability can contribute heavily to health inequalities. Particularly relevant are areas with high cumulative burdens and high social vulnerability termed as “hotspots”. This paper develops an index-based approach to assess these multiple burdens and benefits in combination with vulnerability factors at detailed intra-urban level. The method is applied to the city of Dortmund, Germany. Using non-spatial and spatial methods we assessed inequalities and identified “hotspot” areas in the city. We found modest inequalities burdening higher vulnerable groups in Dortmund (CI = −0.020 at *p* < 0.05). At the detailed intra-urban level, however, inequalities showed strong geographical patterns. Large numbers of “hotspots” exist in the northern part of the city compared to the southern part. A holistic assessment, particularly at a detailed local level, considering both environmental burdens and benefits and their distribution across the population with the different vulnerability, is essential to inform environmental justice debates and to mobilize local stakeholders. Locating “hotspot” areas at this detailed spatial level can serve as a basis to develop interventions that target vulnerable groups to ensure a health conducive equal environment.

## 1. Introduction

People living in urban environments are increasingly exposed to severe conditions of air pollution, noise nuisance, and heat stress which critically influence their health and wellbeing [1]. On the other hand urban areas also provide resources such as parks and green areas that have the potential to attenuate noise levels [2], absorb air pollution concentration [3], regulate microclimate ecosystem services [4] or provide opportunities for health promoting physical activities of their inhabitants [5]. These pathogenic factors (chemical/physical stressors) and salutogenic factors (resources) exist simultaneously and even interact with each other [6,7].

In addition to the co-occurrences of pathogenic and salutogenic factors, urban environments have populations with varying social vulnerability. Social vulnerability describes the way in which various structural factors create the social fabric in which individuals and groups are differentially susceptible to environmental hazards, thereby shaping their ability to cope, adapt or resist differently when exposed to the same environmental hazard [8,9]. Various studies are uncovering the heightened vulnerability among population who belong to racial or ethnic minorities or are of low socio-economic status (SES) [10,11,12]. These socio structural factors, such as race, ethnicity, SES, etc., are found to be determinants of social vulnerability and to be directly related to health effects [13,14] or to act as “effect modifiers” [15,16]. Given a certain level of environmental hazard, groups of a lower SES may bear more health effects than their counterparts. Several reasons have been proposed for widespread environment-related health inequalities among various social structural groups. As noted by Hermans [17], people with a higher SES have more opportunities to adapt to the actual environmental situation; they have more choice about where they live, are able to influence decision making about their locality, and are able to get more involved in planning decisions affecting their living environment [18] (p. 106). On the other hand, the poor and minorities tend to possess less political power to fight the location of environmental hazards in their proximity [19] in [20]; their residential choices are strongly constrained by limited economic resources [21]; they might experience discrimination in the housing market, which increases residential segregation. Studies have reported residential segregation as one such critical link between social structural factors and environment related health inequalities [10,22]. Hence, differential exposure to multiple environmental burdens and benefits and varying levels of social vulnerability across the population may cause serious health inequalities [23].

A holistic assessment, which considers environmental burdens and benefits as well as their distribution across population with different vulnerability, is essential to inform environmental justice debates, to mobilize local stakeholders, and to develop interventions at local level that target vulnerable groups to ensure a health-conducive equal environment [12,24]. Environmental justice-framed studies have explicitly approached the multidimensional relationship between environmental inequalities and social disparities in their distribution [25,26,27]. These studies have a long tradition in the U.S. and the UK and have focused mostly on race, ethnicity and SES, largely ignoring the role of migration. The need to address these environmentally related health inequalities has recently been put on the agenda in many other European cities, focusing predominantly on single environmental variables and their social disparities [2,28]. Along with the efforts to extend the environmental justice investigation to other European cities, there is now a growing recognition that a background of migration can be a social determinant of health and poor SES might itself be a result of migrant status and ethnic origin because of various processes of social exclusion, residential segregation as well as employment segregation [29]. Koeckler [30] included, while exploring the role of social vulnerability and household coping strategies for Kassel in Germany, the percentage of foreigners in an index of SES in addition to the proportion of children and teenagers and unemployment rate. In the environmental justice study for Kassel, the socio-economic variables were measured as income, educational attainment, migration background, and number of children [31]. The author concluded that the households with lower SES and a background of migration to Germany live disproportionately in districts with lower environmental quality. Considering foreigners and welfare recipients as disempowered segments of population with minority status and poverty, Raddatz and Mennis [20] found that industries and toxic release facilities are disproportionately located within and closer to neighborhoods with a relatively higher proportion of foreigners and poor people in Hamburg, Germany. Following the historical influx of working class immigrants, the authors [20] speculated that persistent labor segregation, in combination with discrimination in housing, and lack of political empowerment to prevent the siting of environmental burdens, have reinforced residential segregation to produce patterns of environmental inequalities. In the German context, it is however to be noted that the status of foreigners or the background of migration is different from the characteristics of race or ethnicity as they are used in the U.S. context. Although the immigrants from the 1950s and 1960s who have resided in Germany for many years have acquired German citizenship through naturalization, and many children of those immigrants born in Germany have been granted German citizenship, they still bear the status of having a migration background [20]. Besides, existing studies have used administrative boundaries such as census tracts and districts that are often large and aggregated, in demonstrating inequalities with respect to environmental factors across various SES groups. However, there is growing evidence that substantial inequalities also exist on smaller and local scales [32]. Therefore, an uneven distribution of multiple environmental burdens and benefits across areas with different social vulnerabilities remains to be investigated on a small, local scale.

This paper presents an approach to assess multiple environmental features including both pathogenic and salutogenic factors across a population with different vulnerabilities at a local scale. The method includes the construction of an integrated index using a number of environmental indicators that define residential exposure to various environmental aspects, as well as social vulnerability indicators of the residential population at a local scale. The paper describes an application of the method to the city of Dortmund, Germany to illustrate potential applications, complexities and the usability of the method for identifying “hotspots” of inequalities. It is a new approach to assess an uneven distribution of multiple environmental burdens and benefits taking into account the vulnerability of the population at a detailed intra-urban scale. The indices rely on data from the city administration and facilitate stakeholders to monitor the magnitude of these inequalities locally.

## 2. Cumulative Assessment of Multiple Environmental Burdens and Social Vulnerability

Spatial assessment and visualization of geographical areas with different environmental burdens and benefits across social vulnerable groups provide a strong tool in environmental planning and policy formulation and in informing environmental justice debates [33]. Such an assessment when conducted at a detailed scale helps to identify “hotspots” of groups and areas within the city that are most susceptible to harm due to the exposure to multiple environmental factors that might cause health inequalities considering the vulnerability of these groups and areas. While most environmental regulations and strategies in Germany are developed at the regional or national level, municipalities are responsible and have the freedom to implement those plans and strategies at local level [34]. Therefore, easily comprehensible assessment methods should enable local agencies to design, implement and prioritize remedial activities as targeted interventions are particularly visible at this scale.

Various tools and methods have been proposed for the assessment of multiple stressors. While single or multiple environmental factors (especially burdens) are increasingly being considered in these studies, approaches including simultaneous exposure to both burdens and benefits along with social vulnerability of the population are rare. Lakes et al. [2] included noise exposure and vegetation as an indicator of burden and benefit respectively to assess environmental justice issues using a social development index at planning unit level for Berlin. Pearce et al. [26,35] calculated a multiple environmental deprivation index (MEDIx) at ward level on a nation-wide scale and showed “triple jeopardy” of environmental burdens and benefits deprivation, socio-economic disparities, and health outcome. Su et al. [36] advocate that their CEHII index is able to aggregate multiple environmental factors and demonstrate its application by assessing inequality across non-white population and poverty (income). Their index focused on air pollution hazards whereas Wheeler [37] focused on air quality, air pollution inventory, landfills, and major accident sites in England and Wales using a deprivation index.

All these methods are specifically being used to assess geographical areas at a regional and national level where the unit of analysis is a strict administrative boundary, except for the work of Sadd et al. [38], who used the neighborhood scale unit, and Vlachokostas et al. [39], who used the activity space of people as a unit of analysis. We argue that these methods are insufficient to provide a small-scaled assessment of multiple burdens and benefits across the social vulnerability of the population. The term “multiple burden and benefit” and “cumulative burdens” is used interchangeably in this paper and indicates the simultaneous occurrence of a number of environmental features at the same time in the same place.

## 3. Study Area

With 570,000 inhabitants, Dortmund is one of the ten biggest cities in Germany and the biggest in the Ruhr District (situated in the federal state of North-Rhine Westphalia). The city’s economy boomed and flourished from the early 19th century to the mid-20th century from coal mining, steel industries, and beer brewing. In the mid-20th century, deindustrialization started, causing radical structural changes in most of the cities in the Ruhr District, including Dortmund. Industrialization and deindustrialization had their impact not only on the employment sector, but also on the spatial structure of the city. During the industrial era the city grew from a moderate size to one of the largest cities in the Ruhr District. Growing industrial production in the city demanded a large number of workers. As a result the city opened its doors to migrant workers from other countries. In order to accommodate these workers within walking distance of these industries, housing was developed in close proximity to the industry locations. Later, deindustrialization brought another structural change to the city. The closing down of many industries led to job losses of the many industrial workers on the one hand and several abandoned and derelict spaces on the other.

At present, the spatial effects of these structural changes are different in the southern and northern part of Dortmund as in many other cities in the Ruhr District with a similar history. While the southern outskirts overall have a lower population density, dominated by single family houses with high average income households, the northern part of the inner city, specifically the districts of Nordstadt and Union, have the highest population density, the highest density of people with a migration background, dominated with houses of five storeys or more, and lower average income households with the highest percentage of people receiving social welfare [40]. These spatial effects are strongly related to the historic development of the region as an industrial region and the particular geological structure of the Ruhr coalfield. In the south, industrialization began in the 1850s, the northern part was industrialized at the end of the 19th century. As such the southern outskirts and central part of the city have undergone extensive urban renewal processes during the course of structural change and are less burdened. In the northern part of the city, which was the working-class area of the 1980s and early 1990s, problems have accumulated: demographic, social and ethnic segregation, and multiple deprivation [41,42].

## 4. Materials and Methods

**Figure 1:**
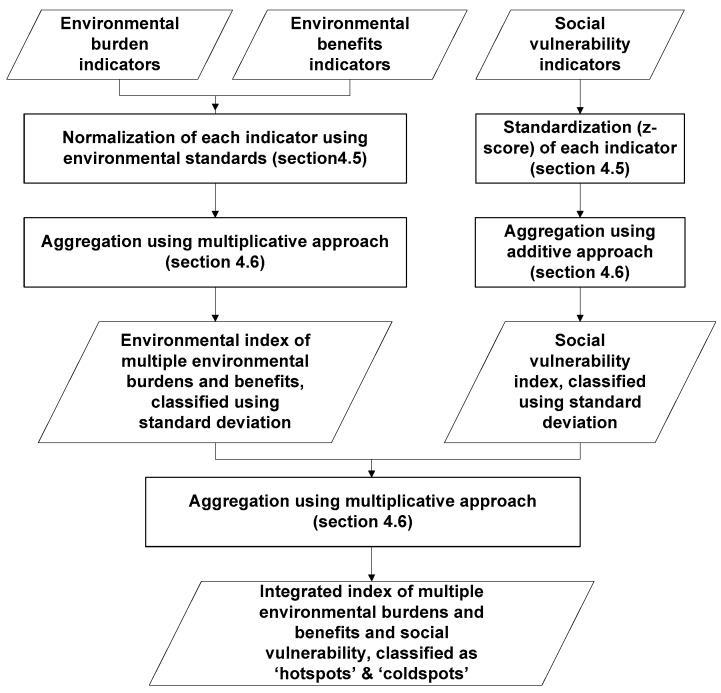
Methodological procedure to develop integrated index of multiple environmental burdens and benefits with social vulnerability of population.

Generally two arguments can be discerned in the construction of an index: (i) a deductive one, based on existing theories and frameworks [35,43]; and (ii) an inductive one, that is data driven without an a priori model where a statistical model explains the observed outcome [44,45]. According to Hinkel [46] there exists a third argument which is based on individual or collective value judgements known as a normative argument. Existing frameworks and scientific knowledge in deductive arguments on multiple environmental burdens and benefits are immature. These frameworks and theories provide guidance on selecting potential environmental factors but not for aggregating. An inductive approach, on the other hand, requires sufficient data which may not always be available at the required scale. In this respect we prefer to use a normative argument. In this approach stakeholder-driven knowledge on the local context is incorporated while pre-selecting a suite of indicators for the city and also aggregating the indicators. For instance, environmental stressors prevalent in urban areas such as PM_10_ and NO_2_ are often correlated as these can share the same sources of pollution (e.g., road traffic), which yield multicollinearity issues in an inductive approach [44]. However, these stressors might lead to different health outcomes [47] and therefore need to be included. The methodological procedure for constructing an integrated index of multiple environmental burdens and benefits with social vulnerability is given in Figure 1.

### 4.1. Environmental Indicators and Data Characteristics

The selection of environmental factors and respective indicators to be included is based on the following criteria:

(1) factors plausibly associated with health;
(2) relevance of the factors specific to Dortmund;
(3) comprehensive data for the entire city at adequate spatial resolution;
(4) representative state variables according to Driving, Pressure, State, Exposure, Effect, Action (DPSEEA) framework [48].

A broad scoping of literature was conducted to identify environmental factors potentially having health impacts [2,35,36,45,47,49]. Only those environmental variables that represent external physical, chemical or biological characteristics of the environment were considered, such as air pollution in the neighborhood rather than indoor air quality [26]. These environmental factors and their indicators were then further screened based on the stakeholders’ local knowledge of the relevance of the indicators for Dortmund collected during a workshop (in September 2013, within the project of Jufo-Salus, a stakeholder workshop was conducted. The main objective of the workshop was to identify key environmental indicators that affect the health of the people in Dortmund and are relevant for the city. Stakeholders that participated in the workshop were from the municipality of Dortmund, health department, planning department, local NGO. Around 20 stakeholders participated in the one-day workshop [50]). Only those environmental factors having data available at neighborhood level were included in the index of multiple environmental burdens and benefits as presented in Table 1. For example, air quality indicators might include many pollutants such as PM_10_, SO_2_, NO_2_, and CO, but we included only PM_10_ and NO_2_ in accordance with relevancy for the city as stated by the stakeholders. Other factors, such as, heat stress, accessibility to health services (general practitioner other than hospitals), though acknowledged as relevant indicators for the city, were excluded due to the absence of fine-scaled data. We further sought to minimize the potential overlap between the indicators in order to prevent double counting [51]. As such the indicators for each environmental factor were chosen to correspond to the state level according to the DPSEEA framework. For example, instead of taking into account traffic density, or urban development, which are driving forces that push the environmental process or put pressure on the environment and may also be an indicator of air quality, we included the air pollutant concentrations that represent the state of the environment as indicators of air pollution.

For each of the environmental indicators as presented in Table 1 the datasets were obtained from the City of Dortmund. Table 2 presents the data sources used to capture each environmental indicator and its detail description.

Air quality includes annual averages of nitrogen dioxide (NO_2_) and particulate matter (PM_10_) concentration (expressed in µg/m^3^) which was modelled on a 125 × 125 m resolution using the LASAT dispersion model. The modelling included emission data from individual sources: traffic (cars, trains, air, and ships), industry, and households (domestic heating and small firing, in and outside Dortmund); having a resolution of 1 km × 1 km resulting from different time frames between 2004 and 2012 (for more details on modelling, see [52]).

Noise nuisance from a single source was modelled for the entire city using the noise dispersion model following the Noise Mapping Decree (Federal Emission Protection Decree and Federal Emission Control Act) and the Noise Mapping Directive 2002/49/EC (Environmental Noise Directive). The following noise level data were acquired: road traffic, industries, above-ground subway, and tram. The noise model data provide a weighted noise pressure level (topography, building footprints, and height were included in the model) for each point location at a 10-m interval (for more details, see [53]). The metric that was used to characterize noise at each point location from an individual source is the European standard L_den_ indicator (day-evening-night level, measured in decibels, dB), an assessment of daily exposure over a 24-h period taking into account residents’ increased sensitivity to noise during the evening (1800 h till 2200 h) and at night (2200 h till 0600 h) [53].

Green spaces were extracted from a land-use data set on existing land-use from the City of Dortmund. The land-use data were prepared using 1:5000 scale aerial photographs from 2013. The data contained detailed land-use categories at parcel level. For green spaces we took public green spaces where access is unrestricted (e.g., parks, cemeteries, green areas, and forest areas). We excluded backyard gardens and urban gardens from green areas as they are private and not accessible to the general public. For the forest we considered areas that are designated as deciduous, coniferous or mixed forest from the land use dataset.

### 4.2. Social Vulnerability Indicators and Data Characteristics

Table 3 shows the list and description of the social indicators that represent the social vulnerability of people in Dortmund. The selection of the social vulnerability indicators was primarily based on the studies specific to the context of Germany [2,20,30,31]. The initial list for SES variables included income, unemployment, education attainment, and migration background. Instead of measured income and unemployment rate we used population receiving public assistance (social welfare known as SGB II (working age population receiving assistance) and SGB XII (basic security for old age, disability, assistance for livelihood share)) as an indicator of unemployment and those living below the poverty line respectively specific to the context of Germany. Based on the inputs from stakeholders during the workshop and other studies [16,54], we further included indicators for sensitive population such as children aged between 6 to 11 (prenatal and neonatal were excluded as we considered the ages between 6 to 11 to be more exposed to the outside environment) and the elderly above 65 years. Moreover, we only included those indicators that could be obtained from census data at neighborhood level. As such, we could not include the indicator on educational attainment after higher school though it was acknowledged as important for Dortmund.

The data characteristics for each indicator of social vulnerablity from the 2014 survey are presented in Table 4.

### 4.3. Data Conditioning and Spatial Resolution

The descriptive statistics for each environmental indicator on a 125 × 125 m grid cell for the entire city are presented in Table 5.

The air pollution data were acquired on a 125 m raster level for the entire city. For the environmental indicators we chose a 125 × 125 m^2^ grid cell as spatial resolution. This resolution provides enough variation within the sub-district level. The point data on noise level for each source were also interpolated at 125 × 125 m^2^ resolution using Inverse Distance Weighted function in Geographic Information System (GIS) [55] in order to match the spatial resolution of the air quality indicator. In addition, noise levels from single sources were integrated using a logarithmic scale [49].

All green and forest areas with unrestricted accessibility, above 0.5 ha in size and provide areas for recreation and physical activity were included in the analysis [47]. Before excluding areas less than 0.5 ha in size, areas that are adjacent to large areas were merged together as we observed that these small patches are in reality part of the large areas and are only separated by pathways or water bodies inside parks or forests. We calculated “crow flight” distance for each 125 m grid cell to any nearest green area separately for parks and forests, and green areas in total (including both parks and forests) using GIS.

The descriptive statistics for each social vulnerability indicators at the level of neighborhoods are presented in Table 6.

We divided each neighborhood into 25 m × 25 m grid cells (hereafter referred to as social units) in order to show intra-urban variations of social vulnerability indicators. The choice of a 25 m spatial resolution for social units was made after a visual inspection to avoid underrepresentation and overrepresentation of residential areas at neighborhood level. The residential areas were extracted from the land-use dataset that shows detailed categories of existing land use in the city at parcel level. We reproduced each social indicator at 25 m resolution using the polycategorical three-class dasymetric method to distribute population from each census zone [56,57]. Initially, each land use with specific housing characteristics was assigned a fixed proportion of the population. The proportion was subsequently adjusted considering actual land use areas with specific housing characteristics within each source zone (here in neighborhood) [58,59]. As such the total population, accounted for on each neighborhood level, is maintained and distributed to residential areas with specific housing characteristics belonging to the same neighborhood. We used Equation (1) as proposed by Langford [58] for the:

(1) $$d_{\text{cs}}\;=\;\frac{P_{s}}{{Ṕ}_{s}}.d_{c}$$

where c = land cover class or urban density class; s = source zone; $d_{\text{cs}}$ = specific density estimate for class c in zone s; Ps = actual population of source zone s; ${Ṕ}_{s}$ = estimated population of source zone s given by Equation (2):

(2) $${Ṕ}_{s}=\sum_{c\;=\;1}^{C}d_{c}n_{\text{cs}}$$

with $d_{c}$ = initial global density estimate of class c; $n_{\text{cs}}$ = number of pixels of class c in source zone s; C = total number of populated land cover classes or urban development classes.

We used physical housing characteristics based on the categories in the land use dataset in terms of number of storeys (two, three, more than five) and land use classes (residential and mixed land use). Occupancy rates and population density in an area varies with socio-economic factors such as wealth, age groups, and cultural differences, and are assumed to be related to physical housing characteristics [58]. The initial global density ($d_{c}$) for each class C was estimated, based on the stakeholders’ opinions regarding residential land use with two-storied (15%), three-storied (30%), more than five-storied buildings (40%), and residential mixed land-use (15%). The descriptive statistics for each social vulnerability indicators on the level of 25 × 25 m^2^ resolution are presented in Table 7.

### 4.4. Normalization or Standardization of Indicators

The steps that are generally addressed before aggregating the indicators are normalization or standardization of each indicator to overcome the incommensurability of the units in which single indicators are measured, followed by weighting of indicators to assign preference of one indicator over the other. We used environmental standards as thresholds for normalizing each environmental indicator. Environmental standards are used in planning and policy making as an acceptable limit that no intervention should exceed. Normalizing the indicator in such a way helps stakeholders to identify areas that have high exceedance of environmental standards. We calculated the ratio value for each grid cell (125 m × 125 m) by dividing the indicator value by the corresponding environmental standard (Table 8). All environmental burdens and resources are transformed in such a way that values less than 1 refer to low burden or good access to resources with respect to the threshold standard, and values higher than 1 to higher burden or poor access to resources. For social vulnerability indicators we used standardization (z-score) to avoid the score being influenced by a high or low value for one variable and to put each variable on the same scale, centered around the city’s mean [60]. Social units (25 m × 25 m) with a positive standardized score show higher level of vulnerability. On the contrary, a negative score shows less vulnerability.

### 4.5. Aggregation of Indicators into Index

The final step of constructing the index is the aggregation of indicators into one composite score. The most commonly used method is the additive or weighted arithmetic mean [2,16,25,54] and the multiplicative or geometric mean method [36,63]. Both methods produce meaningful results in the case indicators are normalized before aggregation as these become dimensionless [63]. Nonetheless, aggregating indicators arithmetically based on equal weights implies that variables are perfect substitutes, which means that a low value in one variable can be compensated by a sufficiently high value in another [39,64]. An implicit assumption of the additive approach is that variables are preferentially independent and non-interactive. Alternatively, the multiplicative approach suggests that a low value in one dimension cannot be fully substituted by a high value in another dimension. This approach is better when it is known that the aggregated entities influence each other [64,65]. Therefore, even if the exact interaction of the environmental indicators are not known and not within the scope of this study, we argue that neighborhoods having higher burdens should not be fully compensated by higher benefits. We therefore used a multiplicative approach to aggregate environmental variables (Equation (3)). The index score for the cumulated multiple burdens and benefits ranges between 0.13 to 1.41. The additive method was used to construct the social vulnerability index (Equation (4)), implying that there can be a compensation between a high and low value. For instance, a neighborhood with a higher number of migrants may not be vulnerable given that they have sufficient resources to escape from environmental burdens. In both indices equal weighting was considered for all the indicators. The score for social vulnerability index ranged between −4.17 to 45.54. We used the standard deviation based visualization for both the environmental and the social vulnerability index to identify areas that have the highest deviation from the city’s mean.

(3) $${\text{EI}}_{j}=\overset{n}{\underset{i\;=\;1}{\prod }}{\left(w_{i}r_{\text{ij}}\right)}^{\frac{1}{n}}$$

where, EI = Environmental Index of multiple environmental burdens and benefits; j = spatial unit, here 125 m × 125 m grid cell; i = environmental indicators under consideration (i = 1, 2, 3,……, n); $r_{\text{ij}}$ = ratio value with respect to indicator i at spatial unit j; $w_{i}$ = weight assigned for each indicator; here it is equal to 1 as equal weight

(4) $${\text{SVI}}_{ĵ}=\sum_{k\;=\;1}^{m}w_{k}p_{kĵ}$$

where, ${\text{SVI}}_{ĵ}$ = Social Vulnerability Index; ĵ = Social units, here 25 m × 25 m grid cells, which fall into j; k = social vulnerability indicators under consideration (k = 1, 2, 3,……,m); $p_{kĵ}$ = standardized score with respect to indicator k at social unit ĵ; $w_{k}$ = weight assigned for each indicator; here it is equal to 1 as equal weight.

An integrated index score of multiple environmental burdens and benefits with social vulnerability was calculated for each social unit (25 m × 25 m). The score was obtained using a multiplicative function (Equation (5)) considering social vulnerability as “effect modifier”.

IESVI = EI × SVI
(5)

where, IESVI = Integrated environmental and social vulnerability index; EI = Environmental Index of multiple environmental burdens and benefits; SVI = Social Vulnerability Index.

Given a similar environmental quality, areas with higher vulnerability suffer greater impact than those with lower vulnerability. The score for integrated environmental and social vulnerability index ranges between −3.8 to 38. In order to visualize the “hotspots” (having high cumulative burdens and high social vulnerability) and “coldspots” (having low cumulative burdens and low social vulnerability) the index value was categorized based on the standard deviation value. Areas with a score of more than 2.5 standard deviation from the city’s mean in the positive direction are termed “hotspots” whereas areas with a score of less than 0.5 standard deviation in the other direction are considered as “coldspots”.

## 5. Results

**Figure 2:**
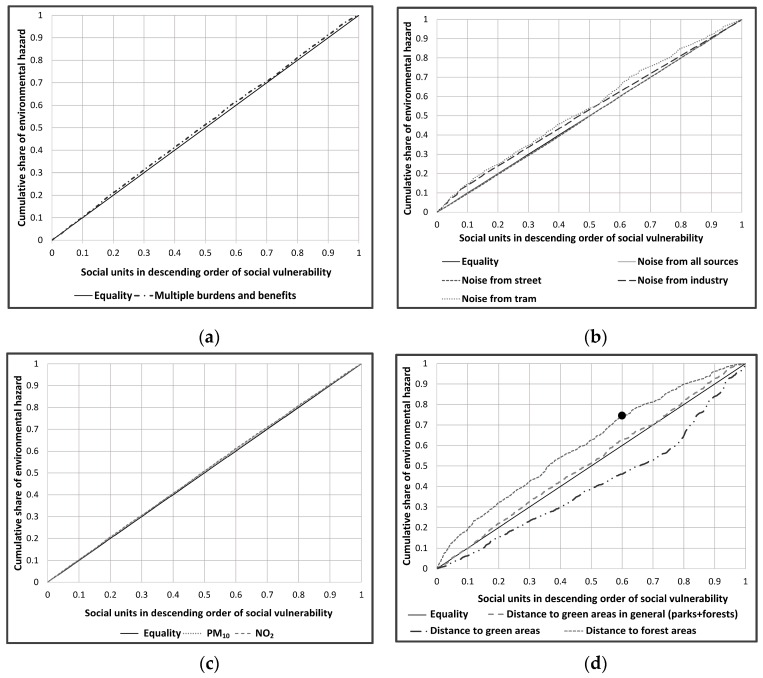
(**a**) Concentration curves illustrating the distribution of indicators for combination of factors with regard to social units having varying social vulnerability; (**b**) Concentration curves illustrating the distribution of noise indicators from individual sources and combined noise exposure (logarithmic addition of noise level from all sources) with regard to social units having varying social vulnerability; (**c**) Concentration curves illustrating the distribution of air pollution indicators with regard to social units having varying social vulnerability; (**d**) Concentration curves illustrating the distribution of green areas (public parks, cemeteries) and forests separately and green areas in general (parks, cemeteries, forests) with regard to social units having varying social vulnerability. For example the point in (**d**) illustrates that when the cumulative share of social units is 60%, those social units with higher social vulnerability bear the disproportionate share of distance to forest areas of 75%.

Concentration curves illustrating the distribution of single and multiple environmental burdens and benefits with regard to social vulnerability scores in social units across the entire city are presented in Figure 2. We constructed the concentration curve by ordering each social unit across the x-axis from highest to lowest in terms of social vulnerability [16,36]. If there is perfect equality in the distribution of environmental burdens and benefits across social vulnerable groups, the graph will be a straight diagonal crossing the origin at 45°. Curves above this equality line indicate an unequal distribution with social units of higher vulnerability bearing disproportionately higher environmental burdens; for curves below the equality line it is the other way around. The farther the curve is above the line of equality, the more concentrated environmental burdens are among social units with higher vulnerability and vice versa.

The concentration curve for multiple burdens and benefits with respect to social vulnerability (Figure 2a) lies almost on top of the equality line. Based on this observation we can assume that there is no pronounced inequality. Exploring the concentration curves for every single environmental factor separately in Figure 2b–d, we observe that all the concentration curves lie rather close to the line of equality, except for access to green areas and access to forest areas, indicating an only limited inequality with respect to individual environmental factors as well. Among all the environmental factors lying above the equality line, access to forest areas shows relatively large deviations followed by noise exposure from tram and industry. On the other hand, among those lying below the equality line, access to green areas affects the social units having lower social vulnerability stronger.

The concentration indices (CI) provided in Table 9, calculated after Kakwani et al. [66], depict both the strength of the relationship between social vulnerability and environmental hazards and the degree of variability in the environmental factors. The CI values range from −1 to 1, with zero indicating equality, negative values indicating environmental burdens disproportionally affecting social units that have a higher vulnerability, and positive values the opposite. We tested the null hypothesis CI = 0 and the significance of the CI index by calculating the standard error, and 95% confidence intervals [66].

Concentration indices and their 95% confidence intervals in Table 9 show that although the degree of inequality with respect to multiple burdens and benefits is very modest, there is a significant relationship between social vulnerability and cumulative burdens and benefits and that social units with higher vulnerability are affected.

Among the single environmental factors with negative CI values (Table 9), access to forest areas is the highest followed by tram and industry noise exposure and NO_2_, all values being statistically significant (*p* < 0.05, see Table 9), i.e., social units with a higher social vulnerability have lower accessibility with respect to forest areas and are also more exposed to tram and industry noise exposure and NO_2_ concentration. Similarly, the relationship between access to green areas (including both forests and parks) and social vulnerability is also significant (*p* < 0.05, see Table 9) suggesting that social units with higher social vulnerability have a lower level of accessibility to resources. On the other hand noise exposure from street and noise exposure irrespective of the source (logarithmic addition) have the least CI value which is positive and significant (*p* < 0.05, see Table 9), suggesting that social units with lower social vulnerability are to some extent exposed to noise pollution. Thus, though less pronounced, there is some degree of inequality that is burdening social units with a higher social vulnerability and it is generally greater with respect to environmental resources than to environmental burdens.

**Figure 3:**
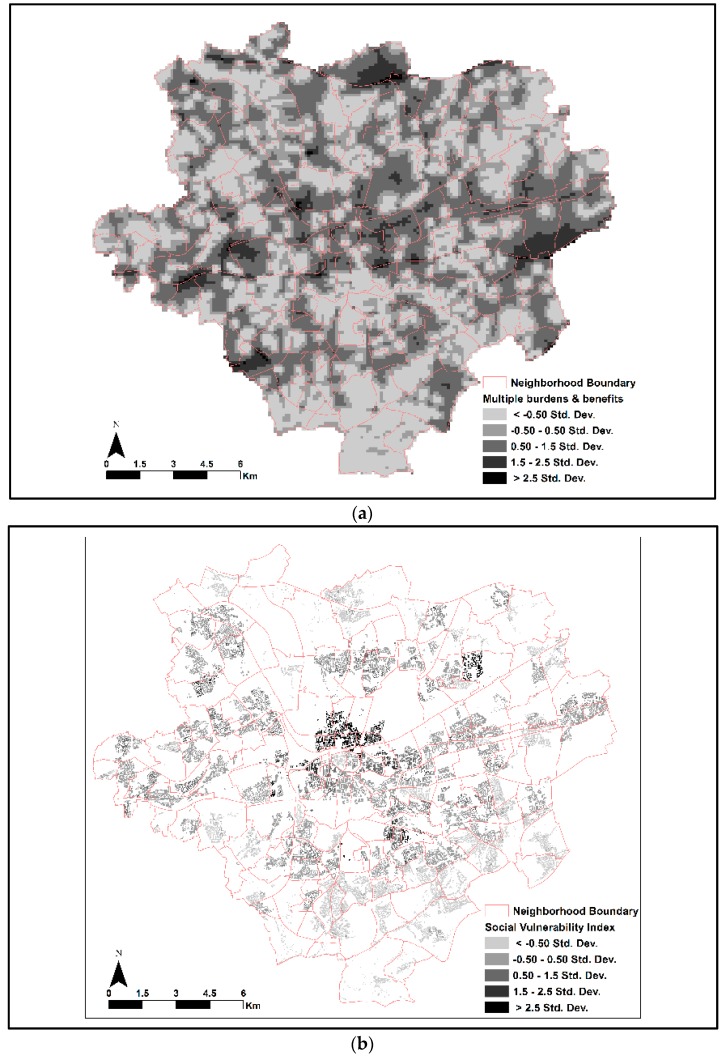
(**a**) Spatial patterns of index score for multiple environmental burdens and benefits in Dortmund; (**b**) Spatial patterns of social vulnerability index score in Dortmund. The positive value of Std. Dev. represents higher cumulated environmental burdens in (**a**) and higher social vulnerability score in (**b**) as compared to the city’s mean. The more negative value of Std. Dev. representing the area has lower cumulated environmental burdens in (**a**) and a lower social vulnerability score in (**b**) as compared to the city’s mean. Finally, the social vulnerability based index of multiple burdens and benefits presented in Figure 4 allows us to identify the spatial pattern of inequalities across the city and enable us to locate “hotspots” (>2.5 Std. Dev.) and “coldspots” (<−0.5 Std. Dev.) of inequalities, i.e., social units with double burdens of low environmental quality and higher social vulnerability or vice versa.

Spatial patterns of cumulated multiple burdens and benefits as illustrated in Figure 3a show a perforated and scattered pattern throughout the city. Smaller clusters with a higher cumulative score compared to the city’s mean (>1.5 Std. Dev.) are found predominantly around the inner city, but in the central eastern and western part of the city too. Similarly, large clusters with a higher cumulative score can be found in the northern and southernmost part of the city, and near the airport region on the eastern city boundary. Unlike the environmental index, the social vulnerability index in Figure 3b shows only a few distinct geographic concentrations of higher vulnerability scores (>2.5 Std. Dev.). The visual inspection of the spatial distribution of vulnerability scores shows that social units with higher vulnerability (>2.5 Std. Dev.) are concentrated within the city core, mostly in the district of Nordstadt. Nevertheless, we observe relatively higher vulnerability scores in the north eastern part of the city. Additionally, there are a few smaller clusters with medium vulnerability levels (1.5–2.5 Std. Dev.) in the southern urban core of the city. High scores are typical in areas with population characterized by a high density of migration background and receiving unemployment benefits.

Large clusters of “hotspots” appear in the northern part of the city center in the district of Nordstadt and in the north-eastern part of the city. Only a few small clusters appear in the southern part of the city. Without exception, the “coldspots” or the social units with both favorable environmental quality and lower social vulnerability are found in the outskirts of the city. We observe that such clusters are located prominently in the urban fringe, mostly in the southern part of the city. Nevertheless, one large “coldspot” can be found in the southern part of the urban core in the downtown area and another one near to the “hotspot” in the north east part of the city. These results can be explained by the fact that these areas have a relatively lower population density. Overall most social units across the entire city have both environmental quality and social vulnerability marked as medium to low or low environmental quality with low social vulnerability. It explains the lower degree of inequalities.

## 6. Discussion

### 6.1. Spatial Inequalities and “hotspots” of Multiple Burdens and Benefits with Respect to Social Vulnerability in Dortmund

**Figure 4:**
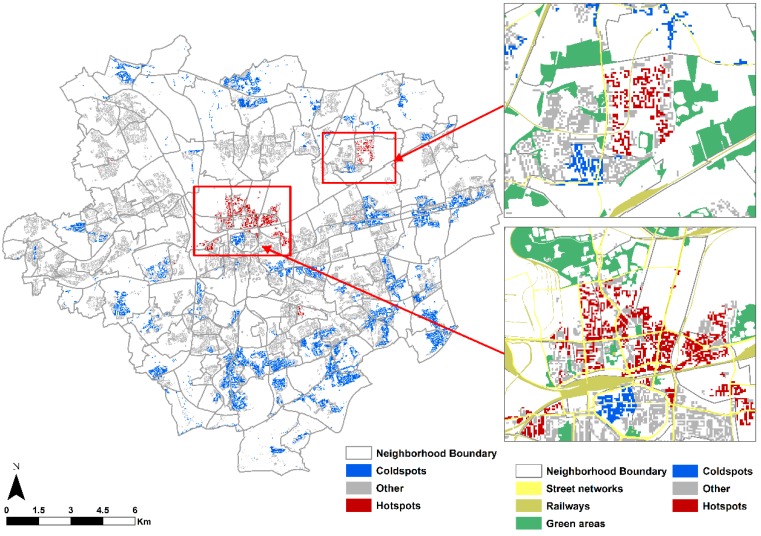
Spatial distribution of “hotspots” and “coldspots” of integrated index of multiple environmental burdens and benefits with social vulnerability.

Our findings suggest that there is only a limited degree of inequality with respect to social vulnerability in Dortmund for both single and multiple environmental burdens and benefits. However, the inequality is statistically significant, demonstrated by CI values at 95% confidence intervals, and is burdening communities with a higher vulnerability. The modest environmental inequality in Dortmund is comparable to European cities found in other studies that also detected a small degree of inequality burdening lower SES groups [2,18]. Consistent with these findings Figure 4 shows some geographical pattern that confirms results of other studies [37]: a relatively high value for environmental burdens in urban core areas coincides with higher social vulnerability index scores producing “hotspots” in the urban core rather than in suburban areas where low to medium value for environmental burdens coincide with lower social vulnerability scores thus producing “coldspots”. 

The concentration indices from the single environmental factors reveal that certain environmental burdens and benefits are more unequally distributed with regard to social vulnerability than others. Inequality with respect to green areas (including both parks and forests) is the highest compared to air pollutants and aggregated noise exposure. However, the CI value (–0.032, *p* < 0.05, see Table 9) suggests that the strength of inequality is rather small. Findings from other studies with respect to this issue reveal divergent results; while some studies found more open spaces in affluent communities than in SES communities [67], other studies showed a more equitable distribution across neighborhoods [68]. In Dortmund, such a low degree of inequality can be attributed to the spatial distribution of green areas and forest areas. As depicted in the spatial distribution maps of access to green areas and forests (Figure S1 provided in supplementary data) the urban core tends to have better accessibility to green areas due to the presence of some of the largest parks such as Fredenbaumpark, Hoeschpark, and Nordmarkt near to the areas where communities with a higher vulnerability are resided. On the other hand suburban areas are closer to forest areas. 

Amongst the environmental burdens, the study shows that inequality with respect to the noise exposure from individual sources (tram and industry) is the highest and is disproportionately burdening communities with a higher vulnerability. The CI values for tram noise exposure (–0.084, *p* < 0.05, see Table 9) and industry noise exposure (–0.049, *p* < 0.05, see Table 9) depict a relatively greater strength as compared to other environmental burdens and are burdening communities with higher social vulnerability. The reason can be attributed to spatial patterns of tram lines following radial patterns and location of industries, both dominating the urban core (Figure S2a,b provided in the supplementary data). Street noise, however, is found ubiquitous and shows a scattered pattern across the entire city with a higher concentration along the major roads and highways (Figure S2c provided in the supplementary data). It is consistent with the findings in other studies where people living in advantaged areas exposed to higher levels of road traffic noise than in disadvantaged areas [2,69], which explains the low positive CI values (0.006, *p* < 0.05, see Table 9) for exposure to street noise. CI value (0.004, *p* < 0.05, see Table 9) with respect to aggregated noise exposure from all the sources was found to be least and affects social units with lower vulnerability indicated by its positive value. However, for Dortmund we need to make the reservation that the noise indicators in this study do not include noise exposure from long distance trains that pass through the city in the east west direction close to the district of Nordstadt in the north and downtown area in the south and noise exposure from the airport on the eastern border of the city. Including these would have implications on the total noise levels and consequently the inequality results. Regarding air pollutants (PM_10_ and NO_2_), they are also disproportionately burdening communities with a higher vulnerability, and inequalities with respect to these pollutants are also significant. This finding confirms results of many other studies showing that communities with lower SES are generally exposed to higher levels of air pollutants more than affluent communities [20]. Nevertheless, the degree of inequality in Dortmund is rather small (CI for PM_10_ = –0.009, *p* < 0.05; CI for NO_2_ = –0.014, *p* < 0.05; see Table 9) that can be attributed to spatial patterns of these air pollutants similar to street noise exposure (Figure S3 provided in the supplementary data). Whilst higher values of pollutants exist in urban core areas where communities with a higher social vulnerability tend to live, it also follows a linear pattern along major roads and highways in the urban fringe where communities with lower vulnerability live. 

Our integrated index of multiple burdens and benefits and social vulnerability demonstrates a strong geographical pattern of inequalities with small scaled variations (Figure 4). Relatively high index scores are located predominantly in the northern part of the urban core particularly in the neighborhoods of Nordstadt and north-eastern part of the city termed as “hotspots”, and areas with lower index values appear in the urban fringe, but also in small clusters in the southern part of the urban core in the neighborhoods of City-West. Why some urban residents benefit from the geographical distribution of environmental burdens and benefits while many others do not has been discussed extensively in literature [70]. The studies emphasized the role of past and present structural processes through which the political economy of different places can act to create local environments and influence the distribution of people, which ultimately result in the observed inequalities [71]. Such processes include historical patterns of industrial development, labor markets, suburbanization and segregation, and economic restructuring. The long history of industrialization and coal mining activity in Dortmund is the most plausible explanation for such a pattern as in other cities with a coal mining history [41,42].

### 6.2. Strengths, Limitations and Sensitivity of Integrated Index of Multiple Environmental Burdens and Benefits and Social Vulnerability

Spatial information of multiple burdens and benefits combined with information on social vulnerability at small scales provide a strong tool to identify “hotspots” of population with a higher level of vulnerability suffering from a lower environmental quality. Such identification of “hotspots” enables targeted interventions and measures in order to reduce health inequalities and allows the prioritization of resource allocation from a need benefit perspective [72]. Detailed information on intra-urban variations could support municipalities in deciding how and where to apply scarce resources to reduce exposure for certain subpopulations at risk and to minimize health inequalities.

Development of a comprehensive index is challenging because of the need to make scientific and policy-related choices in the design process [38]. As such, our integrated index of multiple burdens and benefits and social vulnerability has a number of limitations. Foremost, the choice of indicators is subjective and open to discussion. The selection of indices includes those environmental factors that have potential health effects as well as social vulnerability factors that can alter people’s ability to cope with environmental burdens as evidenced from literature, acknowledged by local stakeholders, and enabled by available databases. We recognize that other health-related environmental characteristics exist as well as other social vulnerability factors relevant in the context of Germany, for which we were unable to obtain adequate small-scaled data. Similarly, we derived access/egress from residential locations to nearest park locations based on assumptions that reasonably qualify for providing recreation. Our method considers “crow flight distance” and ignores street network, connectivity, and barriers that have an effect on actual accessibility [73]. In addition to accessibility, the quality of green areas (e.g., attractiveness, cleanliness, safety, environmental quality) affects the actual usage of green areas and therefore needs to be considered [74]. In particular in the case of Dortmund this aspect is important as Bajracharya et al. [75] found that some parks with good access in the neighborhood of Nordstadt are not considered attractive due to several issues of drugs, crimes, and safety.

Most existing approaches for the assessment of cumulative burdens in combination with social deprivation are based on administrative units/boundaries for identifying “hotspots” and inequalities. These studies overlook detailed spatial variations of environmental factors and social characteristics and suffer from the modifiable areal unit problem [76]. We overcome this issue using a grid based approach to construct our indices. Whilst environmental data were readily available for Dortmund in the form of a grid, the smallest spatial unit of social vulnerability data was only available at statistical sub-district level. Using the three class dasymetric method the efficacy of our index was improved by designating where people actually live rather than assuming an even distribution of residents throughout the statistical sub-district. As a result our index was able to show small scaled variations of “hotspots” of double burdens which we consider a strength of our approach. Nonetheless, we acknowledge the criticality of allocating a proportion of population for each land use class based on housing type. Intuitively, urban and rural areas might have a different proportion of people living in various housing types. Similarly, the same proportion that has been used in our analysis for each indicator may not hold true. An empirical survey in each district, both in urban and rural areas, and for each indicator separately, could provide statistical data to derive the proportional density fraction for each land use category [59].

One of the critical points in the development process of indices is the type of aggregation used. The choice of multiplicative approach while combining environmental indicators was guided by the normative argument—multiplicative approach suggests that a low value in one indicator cannot be fully substituted by a high value in another indicator in contrast to the additive approach that allows full compensation. Nonetheless, given the uncertainty of the aggregation approach we conducted a sensitivity analysis using an additive approach and henceforth derived a concentration index for the cumulated environmental burdens. Similar to the result found in using the multiplicative approach, we found inequality with respect to cumulated environmental burdens using the additive approach and it is burdening social units with higher vulnerability as suggested by the CI value (−0.009, see Table 9) significant at 95% confidence interval (−0.009, −0.008, see Table 9). However, the strength of inequality produced using the additive approach is lesser when compared to the multiplicative approach (CI = −0.020, see Table 9). It is generally expected that the multiplicative approach produces a greater difference than the additive approach and aligns with the finding from Su et al. [36].

The choice of standardization and the use of standard deviation as the basis to yield rank for each spatial unit are other critical points in the development process of indices for multiple burdens and benefits with social vulnerability [2,38]. The use of EU standards supports agencies to identify areas exceeding the threshold and to develop targeted interventions. The aggregation of indicators into one index entails loss of information and creates the risk of equalization of different distinct spatial patterns [39]. The multiplicative approach minimizes such compensation to some extent. Different weighting schemes for individual indicators could help to mitigate the effect [36] but due to limited knowledge about the impact of the selected environmental and social vulnerability variables on health outcome, we applied equal weighting. However, the index is flexible enough to accommodate such weighting schemes in the near future through a deliberative process with experts [11].

## 7. Conclusions

We presented an approach for assessing multiple environmental burdens and benefits with social vulnerability at a spatially disaggregated level. In an application we assessed single and cumulative environmental inequalities with respect to social vulnerability of residents across the entire city of Dortmund, Germany, using concentration curves and a concentration index. “hotspots” of inequalities were identified spatially.

We found only modest but nevertheless significant inequalities regarding multiple environmental burdens and benefits with respect to social vulnerability in Dortmund. The integrated index allows us to locate large clusters in the northern part of the city with relatively high scores termed as “hotspots” indicating that these areas have a higher social vulnerability with a low to medium environmental quality, whereas the southern part of the city generally has a lower index score. More useful and interesting is the fact that the integrated index revealed small-scale variations and patterns of “hotspots” that may be concealed at higher spatial levels.

Despite some methodological and data related limitations, our approach enables us to combine environmental burdens and benefits and to investigate intra-urban spatial inequalities with respect to social vulnerability for the first time. Where spatial inequalities are apparent, causality cannot be determined with our analyses. It might be possible that environmental burdens and limited environmental resources in deprived areas have been directed deliberately or that such observed inequalities could be random due to historical industrial development, operation of housing markets, labor markets and subsequent differential migration of those people that can afford to move away from environmental burdens. Nonetheless, “whatever the root causes, if inequality exists, and policies and programs do not act to reduce it, then they act to perpetuate and reinforce environmental injustices” [37]. In this regard an analysis of distributional aspects of environmental injustice could be informative and useful to target remedial activities and to allocate scarce resources to such areas.

Our approach focused on combining multiple environmental burdens and benefits with respect to social vulnerability and did not consider associations with different health outcomes. In this respect further validation of identified “hotspots” with health-related data is needed as in other studies [35]. As Lakes et al. [2] have advocated, the challenge for such analyses is inherent due to data restrictions on morbidity and mortality and this holds true for the city of Dortmund. Moreover, the challenge is further accentuated when analyses require health-related data at finer scales as in our approach largely due to data privacy. A data envelope technique such as geomasking could be useful and needs to be explored further to include health data in such small-scaled analyses while preserving data privacy [77].

Underlying complexity and scientific evidence on whether different environmental factors have synergistic, additive or interactive effects and different pathways, through which multiple environmental factors together with social vulnerability may affect human health, still remain incomplete and in a formative stage [36,39]. Therefore, our approach assumes a simplistic viewpoint of the existence of multiple environmental factors simultaneously at the same place and the same time with social vulnerability as an “effect modifier”. Furthermore, where composite scores can be useful in many situations, a major limitation is that it leads to loss of information about how the different factors that make up the integrated index interact with each other and contribute to the index score [39]. In this regard we want to emphasize on what is referred to as the “danger of simplistic policy conclusions” [64] in [2]. Nevertheless, having more information is intrinsically good to enable an informed and reasoned debate on the situation [27] and such information, when developed in close cooperation with stakeholders, could promote “action-oriented responses to the research findings” [2]. With a number of calls from the scientific community for the need to involve stakeholders from different disciplines to tackle environment related health inequalities [1,78,79] it is warranted that assessment of multiple burdens and benefits including social vulnerability should be able to incorporate both expert and local knowledge. Involving stakeholders in each phase of development and assessment could strengthen the partnership between practice and research and enable the utility of the information in policy and decision-making. However, involving stakeholders from various disciplines is not without challenge as they might have a diverse knowledge and different understandings of cartographic representations as found by Lakes et al. [2]. The challenge is then to facilitate stakeholder participation from various disciplines, to support them in a joint assessment of multiple environmental burdens and benefits with social vulnerability, to provide useful insights on the utility of the information on existing inequalities and “hotspots” in the city, and to discuss how this information can be incorporated in actual planning practice and policy.

## Figures and Tables

**Table 1:** Environmental indicators included in the index of multiple environmental burdens and benefits.

Dimension	Domain	Description of Indicators
Environmental burdens	Air quality	Annual average concentration of PM_10_ (µg/m^3^)
		Annual average concentration of NO_2_ (µg/m^3^)
	Noise nuisance	Noise level from individual sources (industries, street and tram) measured in decibel (dB)
		Logarithmic aggregation of noise level from all sources (dB)
Environmental benefits	Green spaces	Accessibility to green areas >0.5 ha in size within walking distance
		Accessibility to forest areas of >0.5 ha in size within walking distance
		Accessibility to green areas in general (forests, parks, cemeteries) >0.5 ha in size within walking distance

**Table 2:** Data characteristics and sources for environmental factors.

Domain	Data	Data Source	Areal Unit
Air Quality	Average concentration of air pollutants (PM_10_, NO_2_) in a year	City of Dortmund, 2013	Grid (125 × 125 m)
Noise nuisance	Noise level from individual sources (industry, traffic, tram)	City of Dortmund, 2013	Point data at 10 m interval
Green spaces	Land use	Current land use map from City of Dortmund	Parcel level

**Table 3:** Social indicators included in the index of social vulnerabiltiy.

Dimension	Domain	Description of Indicators
Social vulnerability	Sensitive population	Number of people aged between 6 and 11 (persons/625 m^2^)
		Number of older adults aged 65 years and over (persons/625 m^2^)
	Social and economic	Number of people with migration background (persons/625 m^2^)
		Number of people receiving SGB II (persons/625 m^2^)
		Number of people receiving SGB XII (persons/625 m^2^)

**Table 4:** Data characteristics and sources for social factors.

Domain	Data	Data Source	Areal Unit
Sensitive population	Population aged between 6 and 11 years	Social structure Atlas from City of Dortmund, department of Statistic	Neighborhood level
	Population aged 65 years and older		
Social and economic	Proportion of population having migration background		
	Proportion of population receiving SGB II		
	Proportion of population receiving SGB XII		

**Table 5:** Descriptive statistics for environmental indicators.

Indicators	Min	Max	Mean	Standard Deviation (Std. Dev.)
Annual average concentration of PM_10_ (µg/m^3^)	22.08	394.40	24.90	5.45
Annual average concentration of NO_2_ (µg/m^3^)	21.79	116.76	32.06	8.12
Noise level from individual sources (industries, street and tram) measured in decibel (dB)	0 ^a^, 0 ^b^, 0 ^c^	76.16 ^a^, 89.05 ^b^, 69.31 ^c^	2.66 ^a^ , 55.7 ^b^, 5.6 ^c^	10.89 ^a^, 8.6 ^b^, 15.19 ^c^
Logarithmic aggregation of noise level from all sources (dB)	14.7	89.05	56	8.44
Accessibility to green areas >0.5 ha in size within walking distance (m)	1	2053	452.5	388.6
Accessibility to forest areas of >0.5 ha in size within walking distance (m)	1	2011.09	301.00	337.27
Accessibility to green areas in general (forests, parks, cemeteries) >0.5 ha in size within walking distance (m)	1	1344.83	152.80	188.64

^a^ refers to value for industries; ^b^ refers to value for street; ^c^ refers to value for tram.

**Table 6:** Descriptive statistics for indicators of social vulnerability at statistical sub-districts level.

Indicators	Min	Max	Mean	Std. Dev.
Number of people aged between (persons/km^2^)	0	2806.3	166.07	263.97
Number of older adults aged 65 years and over (persons/km^2^)	5.2	2943.63	620.97	515.29
Number of people with migration background (persons/km^2^)	1.05	23,884.80	1142.79	2410.75
Number of people receiving SGB II (persons/km^2^)	0	13711	626.68	1373.31
Number of people receiving SGB XII (persons/km^2^)	0	1243.04	68.11	138.44

**Table 7:** Descriptive statistics for indicators of social vulnerability at the level of social units.

Indicators	Min	Max	Mean	Std. Dev
Number of people aged 6–11 (persons/625 m^2^)	0	4.8	0.42	0.37
Number of older adults aged 65 and over years (persons/625 m^2^ )	0.4	24.4	1.71	0.96
Number of people with migration background (persons/625 m^2^)	0.08	41.5	2.62	3.59
Number of people receiving SGB II (persons/625 m^2^)	0	23.8	1.44	2.06
Number of people receiving SGB XII (persons/625 m^2^)	0	2.81	0.15	0.23

**Table 8:** Environmental standards used to normalize each environmental indicators.

Environmental Indicators	Threshold Values	Source
Annual average PM_10_ concentration	40 µg/m^3^	Deutscher Bundestag [61]
Annual average NO_2_ concentration	40 µg/m^3^	
Annual average noise level	55 dB	EU [62]
Distance to green spaces >0.5 ha	500 m	Honold et al. [47]

**Table 9:** Concentration indices (CI), standard errors (SE) and 95% confidence intervals for individual environmental factors and multiple environmental burdens and benefits with respect to social vulnerability.

S.N	Environmental Factors	CI	SE(C)	Low	High
a	PM_10_	−0.009	0.000	−0.009	−0.010
b	NO_2_	−0.014	0.000	−0.013	−0.015
c	Distance to green areas in general (green areas, forests)	−0.032	0.002	−0.028	−0.036
c-1	Distance to green areas	0.178	0.002	0.175	0.182
c-2	Distance to forest areas	−0.208	0.002	−0.204	−0.211
d	Noise from all sources (logarithmic addition)	0.004	0.000	0.003	0.004
d-1	Noise from street	0.006	0.000	0.005	0.006
d-2	Noise from industry	−0.049	0.001	−0.046	−0.051
d-3	Noise from tram	−0.084	0.002	−0.081	−0.088
	Multiple environmental burdens and benefits (a, b, c, d)	−0.020	0.001	−0.019	−0.022

Note: All the value is significant at 95% confidence interval (*p* < 0.05).

## Supplementary Materials

The following are available online at www.mdpi.com/1660-4601/13/7/691/s1. Supplementary information S1: Maps on individual environmental indicators; Figure S1: Spatial patterns of individual indicator on environmental benefits normalized with respect to environmental standards, Figure S2: Spatial patterns of individual indicators on noise exposure from each source normalized with respect to environmental standards, Figure S3: Spatial patterns of individual indicators on air pollutants normalized with respect to environmental standards. Supplementary information S2: Calculating concentration index, standard error and *t*-test.

## Abbreviations

The following abbreviations have been used:

CEHII	Cumulative Environmental Hazard Inequality Index
CI	Concentration Index
dB	Decibel
DPSEEA	Driving Pressure State Exposure Effects Action
GIS	Geographic Information System
Ha	Hectare
IDW	Inverse Distance Weighted
MEDIx	Mutiple environmental deprivation index
NO_2_	Nitrogen dioxide
PM_10_	Particulate matter
SE	Standard Error
SES	Socio-economic status
Std. Dev.	Standard deviation
