# Supplementary material for: Environmental Health Related Socio-Spatial Inequalities: Identifying “Hotspots” of Environmental Burdens and Social Vulnerability
Source: Int J Environ Res Public Health. 2016 Jul 9;13(7):691. doi: 10.3390/ijerph13070691 (PMC4962232; doi:10.3390/ijerph13070691)
Supplement: Supplementary file 1 [file ijerph-13-00691-s001.pdf]

Supplementary Materials: Environmental Health Related Socio-Spatial Inequalities: Identifying "Hotspots" of Multiple Environmental Burdens and Social Vulnerability

Rehana Shrestha, Johannes Flacke, Javier Martinez and Martin van Maarseveen

Supplementary Information S1: Maps on individual environmental indicators

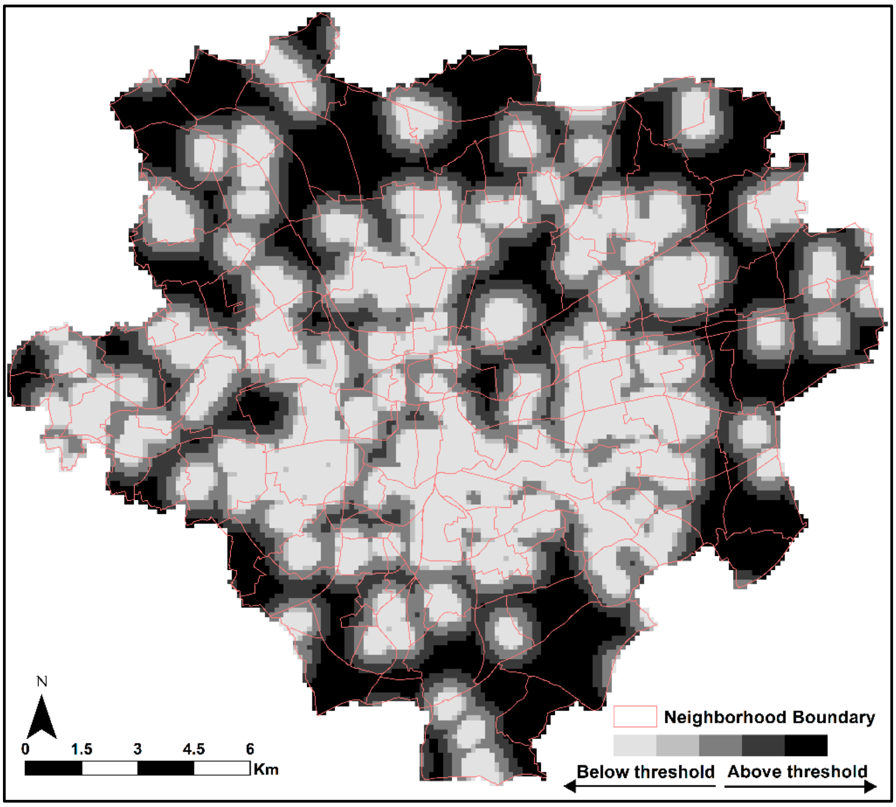

(**a**)

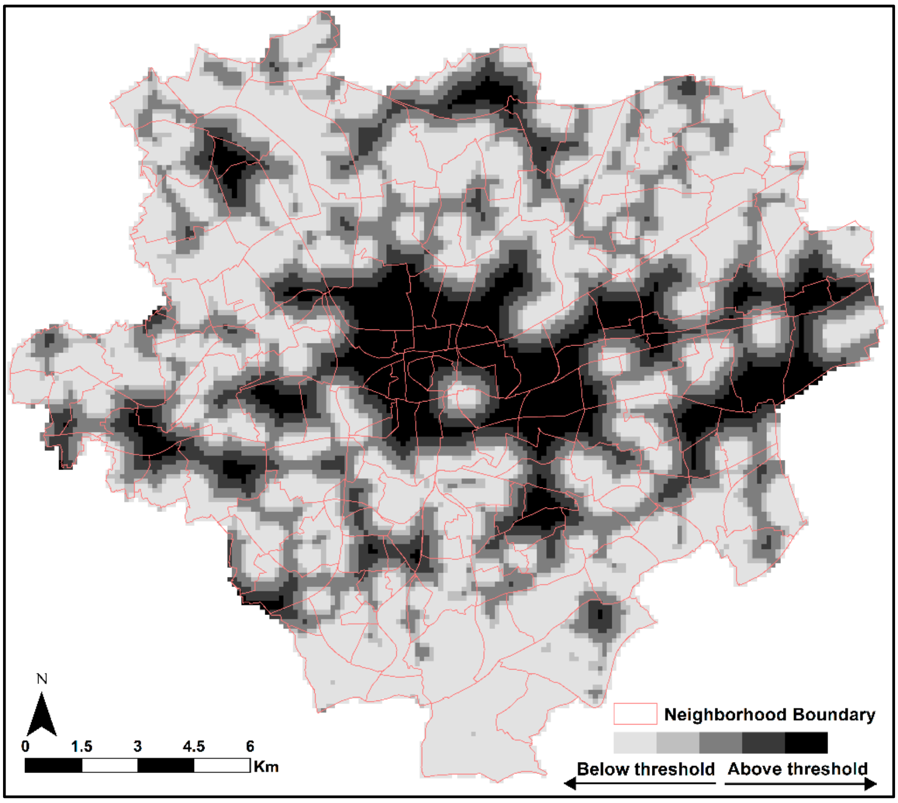

(**b**)

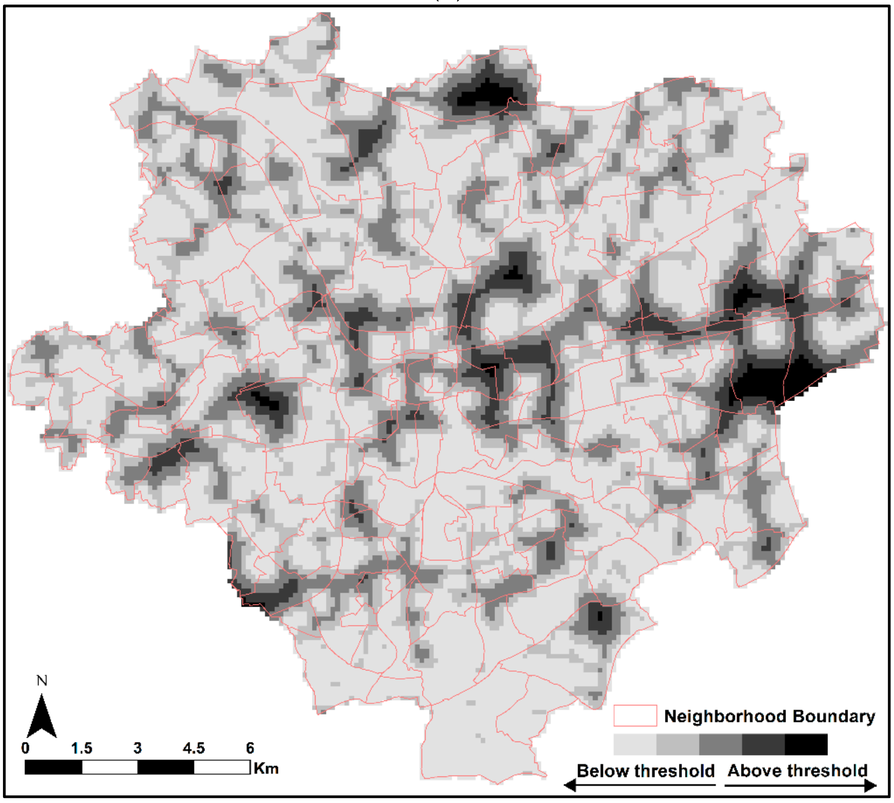

(**c**)

Figure S1. Spatial patterns of individual indicator on environmental benefits normalized with respect to environmental standards (legend shows deviation above and below given threshold value), (**a**) Accessibility to green areas (parks, cemeteries) (**b**) Accessibility to forests (**c**) Accessibility to green areas in general (including parks, cemeteries and forests).

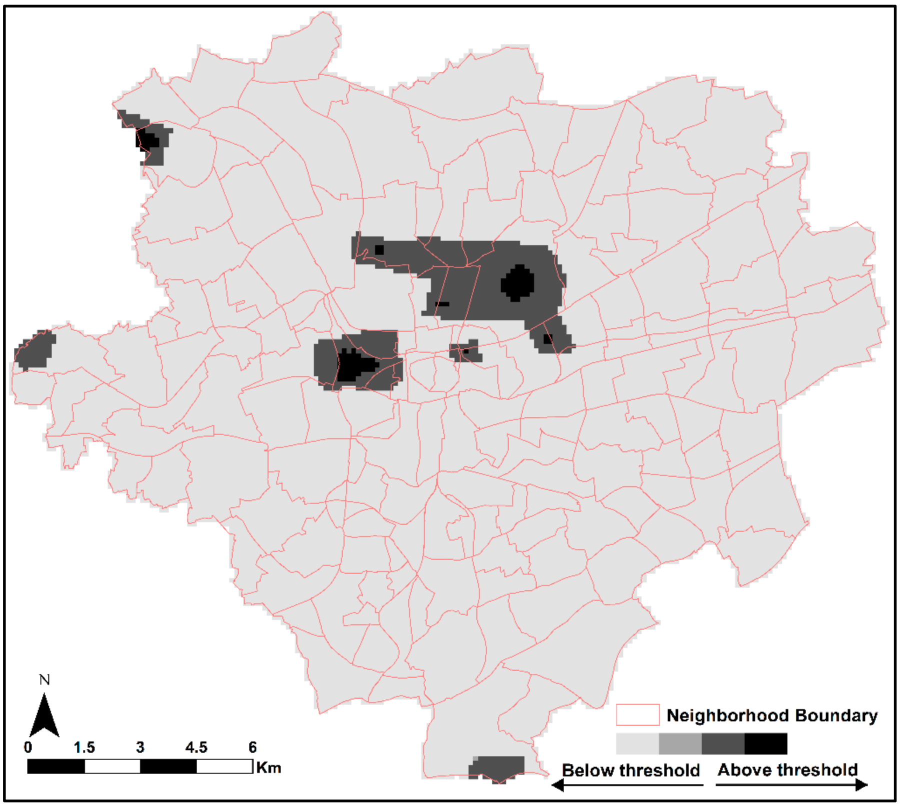
N
0
1.5
3
4.5
6
Km
Neighborhood Boundary
Below threshold
Above threshold

(**a**)

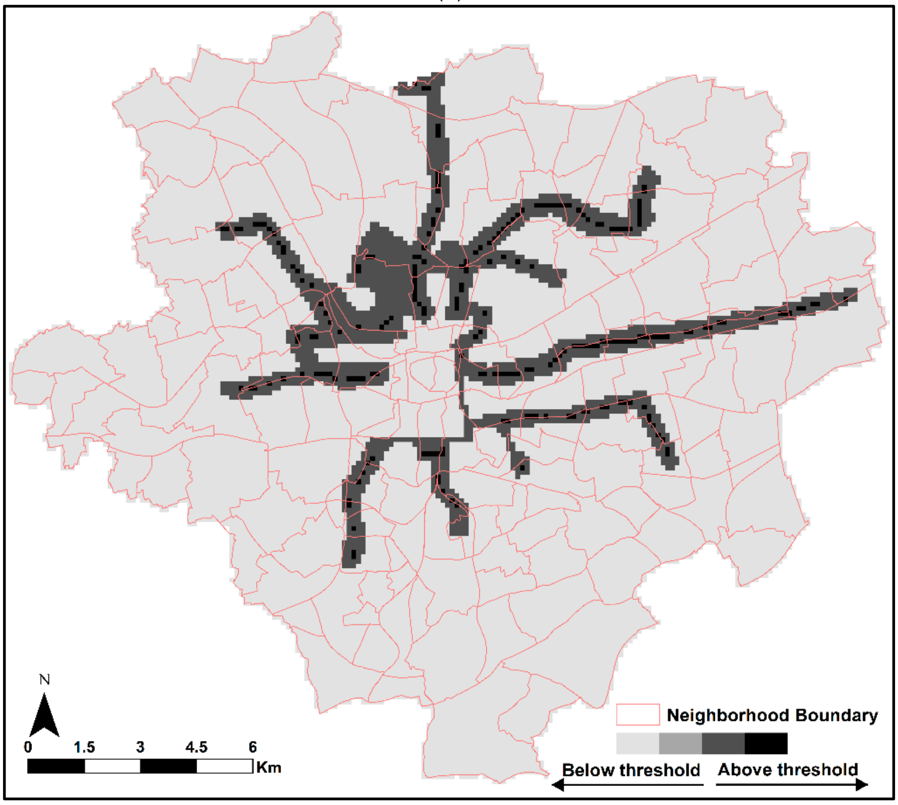
N
0
1.5
3
4.5
6
Km
Neighborhood Boundary
Below threshold
Above threshold

(**b**)

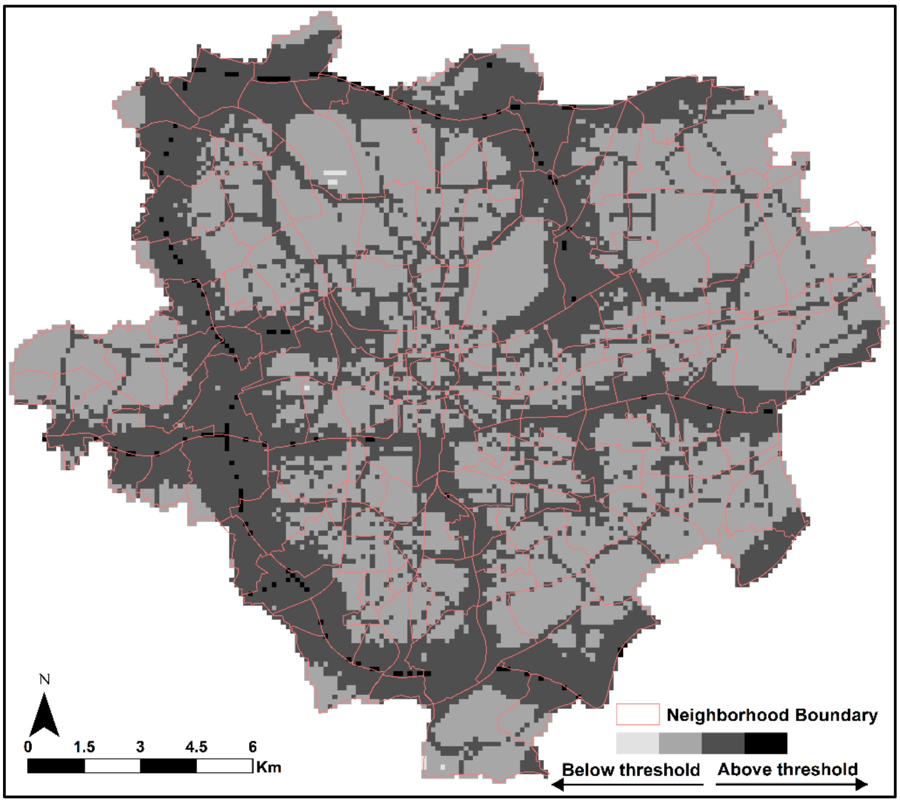

(**c**)

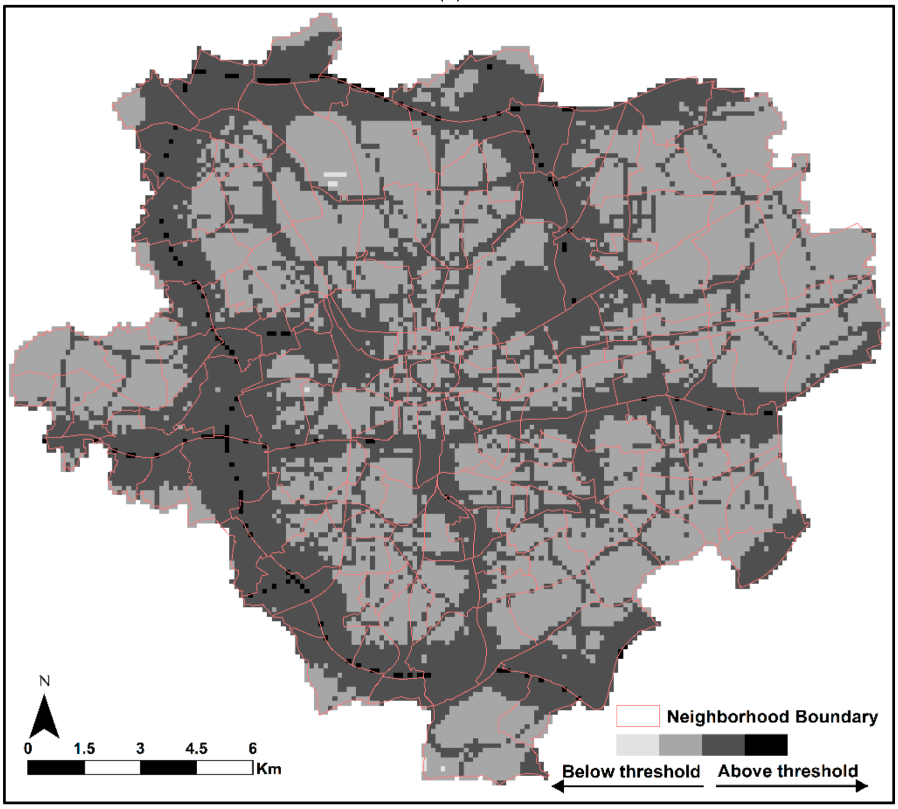

(**d**)

Figure S2. Spatial patterns of individual indicators on noise exposure from each source normalized with respect to environmental standards (legend shows deviation above and below given threshold value), (**a**) Noise exposure from industry (**b**) Noise exposure from trams (**c**) Noise exposure from traffics on major streets and highways (**d**) Combined noise exposure including all three sources (street, tram, industry) using logarithmic addition method.

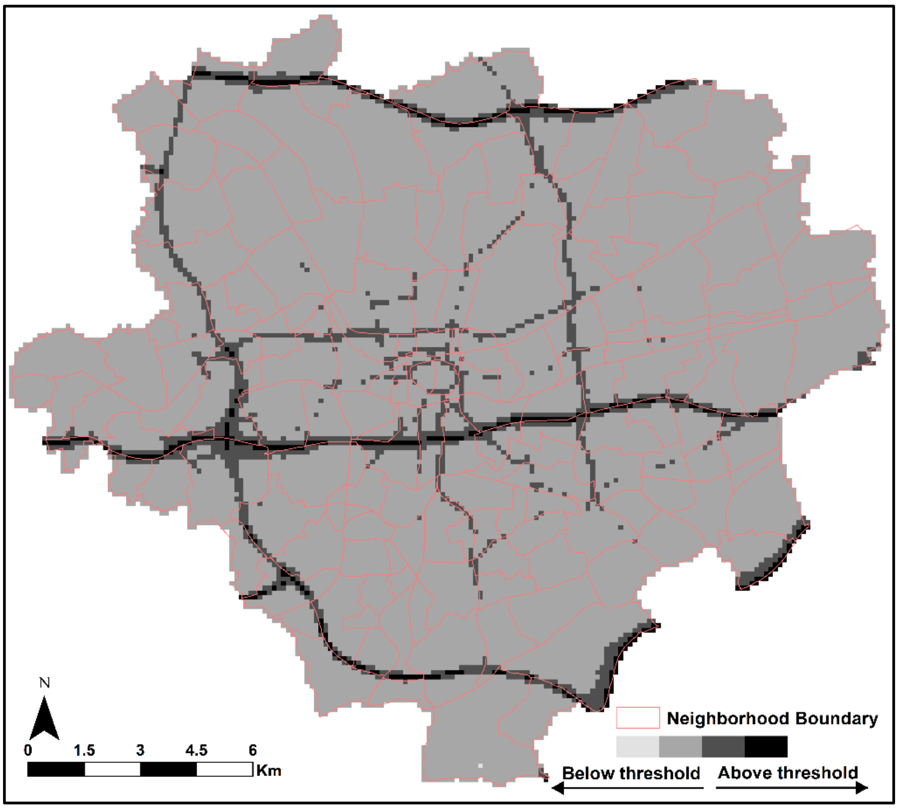

(**a**)

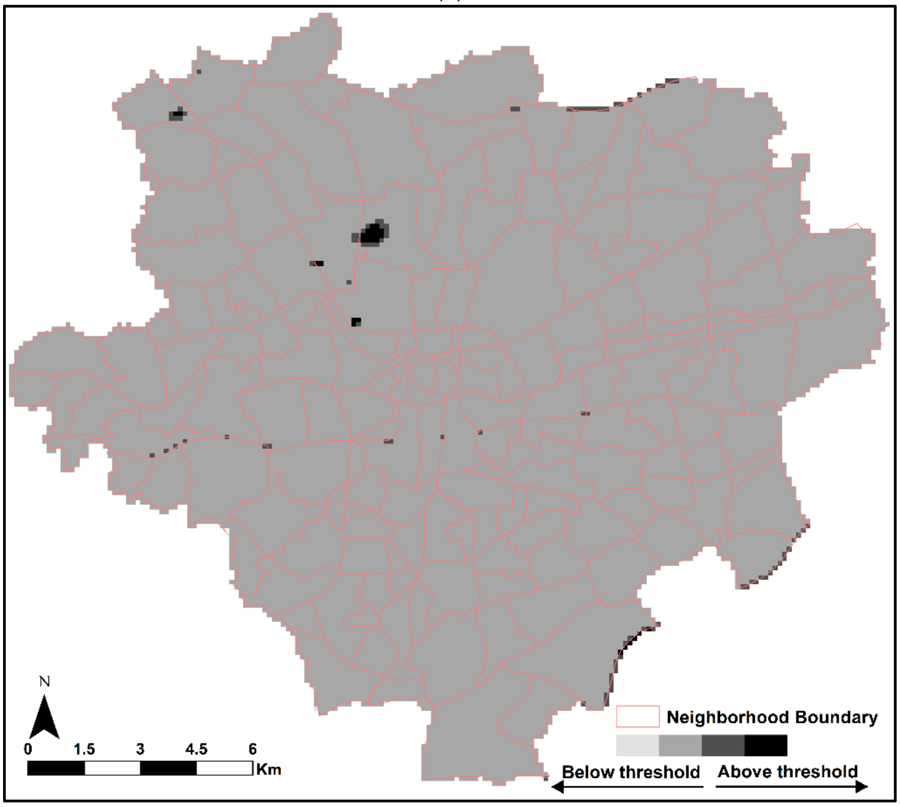

(**b**)

Figure S3. Spatial patterns of individual indicators on air pollutants normalized with respect to environmental standards (legend shows deviation above and below given threshold value), (**a**) Annual average NO_2 concentration (**b**) Annual average PM_{10} concentration.

Supplementary Information S2: Calculating concentration index, standard error and *t*-test for each indicator across social vulnerability

Concentration Index

The concentration index (CI) for individual observations is computed using the following formula by Kakwani et al. [1]

$$\text{CI}=\frac{2}{n\times\mu}\sum_{i=1}^{n}x_iR_i-1 \quad (1)$$

where, n is the sample size, x_i is the indicator of environmental burden for each social unit *i*, μ is the mean of environmental burden indicator, and R_i is the fractional rank in percentage.

Standard Error

A standard error of the estimator of concentration index (CI) can be computed using a formula given in Kakwani et al. [1]. The variance of the estimator of C is given by

$$var(\widehat{CI})=\frac{1}{n}\left[\frac{1}{n}\sum_{i=1}^{n}a_i^2-(1+CI)^2\right] \quad (2)$$

where *n* is the sample size and

$$a_i=\frac{y_i}{\mu}(2R_i-1-CI)+2-q_{i-1}-q_i \quad (3)$$

$$q_i=\frac{1}{\mu n}\sum_{\gamma=1}^{i}y_i \quad (4)$$

is the ordinate of the concentration curve L(*p*), and q_0 = 0.

Calculating t for t-test

In the construction of a concentration index in this paper, the whole social units was taken into consideration, *t* could then be calculated by

$$t=\frac{CI}{\sqrt{var(\widehat{CI})}} \quad (5)$$

References

1. Kakwani, N.; Wagstaff, A.; Van Doorslaer, E. Socioeconomic inequalities in health: Measurement, computation, and statistical inference. *J. Econom.* **1997**, *77*, 87–103.

© 2016 by the authors; licensee MDPI, Basel, Switzerland. This article is an open access article distributed under the terms and conditions of the Creative Commons by Attribution (CC-BY) license (http://creativecommons.org/licenses/by/4.0/).
